# Systemic inflammatory response in erderly patients following hernioplastical operation

**DOI:** 10.1186/1742-4933-3-3

**Published:** 2006-03-29

**Authors:** Gaetano Di Vita, Carmela Rita Balistreri, Francesco Arcoleo, Salvatore Buscemi, Enrico Cillari, Marcello Donati, Maria Garofalo, Florinda Listì, Maria Paola Grimaldi, Rosalia Patti, Giuseppina Candore

**Affiliations:** 1 Sezione di Chirurgia generale, Dipartimento di Discipline Chirurgiche ed Oncologiche, Università di Palermo, Italy; 2 Gruppo di Studio sull'Immunosenescenza, Dipartimento di Biopatologia e Metodologie Biomediche, Università di Palermo, Italy; 3 Laboratorio di Patologia Clinica, Azienda Ospedaliera V. Cervello, Palermo, Italy; 4 Dipartimento di Scienze Chirurgiche, Trapianto e Tecniche avanzate, Università di Catania, Catania, Italy

## Abstract

The number of old and oldest old patients undergoing surgery of varying severity is increasing. Ageing is a process that changes the performances of most physiological systems and increases susceptibility to diseases and death; accordingly, host responses to surgical stress are altered with ageing and the occurrence of age-related increase in susceptibility to post-operative complications has been claimed. Twenty-four male patients undergoing Lichtenstein (LH) hernioplasty for unilateral inguinal hernia were included in this study and divided in two groups (Young and Old respectively), according to their age. As expression of the acute phase response, we measured changes in concentration of pro-inflammatory cytokines Tumor necrosis factor-α and Interleukin-1β, leukocytes, acute phase proteins C-reactive protein and α 1-antitrypsin. Elderly humans showed prolonged and strong inflammatory activity compared to younger subjects in response to surgical stress, indicating that the acute-phase response to surgical stress of elderly humans varies from that of the young, showing initial hyperactivity and a delayed termination of the response. Thus, the acute phase response to surgical stress is higher in old subjects, but the clinical significance of this remains unclear. It is not known whether a causal relationship exists between this stronger acute phase response and the increases in susceptibility to post-operative complications observed in aged patients.

## Introduction

It has been well documented that the immune function declines with age [1]. In the elderly, several alterations in innate and clonotypic immunity have been described [2,3]. In particular, it has been found that increased inflammatory activity accompanies ageing and an increased plasma and serum levels of inflammatory mediators have been observed in old and oldest old individuals [4]. In fact, ageing is a process that changes the performances of most physiological systems and increases susceptibility to diseases and death. Accordingly, host responses to surgical stress are altered with ageing and the occurrence of age-related increase in susceptibility to postoperative complications has been claimed [5]. However, the biological mechanism responsible for increased susceptibility to postoperative complications remains unclear. The surgical stress induces a profound but transient depletion of all types of circulating lymphocytes, which may contribute to postoperative immunosuppression [5]. Besides, in response to cell injury elicited by trauma, as a surgical stress, the inflammatory responses set in, constituting a complex network of molecular and cellular interactions to facilitate a return to physiological homeostasis and to tissue repair. The response is composed both of immediate local events and of systemic activation mediated by cytokines. The pro-inflammatory cytokines such as Tumor necrosis factor-α (TNF-α) and Interleukin (IL)-1β act as the earliest mediators of the acute phase response (APR), which comprises immediate local events at the site of inflammation and activation of systemic features including increased liver-derived acute phase proteins, fever, and neutrophilia. Both cytokines (TNF-α and IL-1β) induce a second wave of cytokines including IL-6 and chemokines [5-8].

The aim of the present study was to examine if the APR, elicited by surgical hernioplastical operation using Lichtenstein (LH) procedure, differed between old and young individuals. It is noteworthy that the involvement of inflammatory mediators in young patients, undergoing LH procedure using polypropylene prosthetic materials or conventional Bassini hernia repair (BH), was higher in LH patients [9,10]. Indeed, the LH induces less pain and more rapid postoperative recovery, but it is associated to a higher inflammatory response compared to BH, likely due to polypropylene mesh [9,10]. So, we measured the changes in concentration of leukocytes, of proinflammatory cytokines, including TNF-α and IL-1β, of acute phase proteins such as C-reactive protein (CRP) and alpha 1-antitrypsin (AAT) in sera from 12 old patients and 12 young patients at preoperative time and at 6, 24, 48 hours postoperatively.

## Materials and methods

### Patients and operative procedures

Twenty-four male patients with unilateral inguinal hernia without complications or recurrence admitted to the Second Surgery Unit of Surgery Department of "Paolo Giaccone" University Hospital of Palermo were included in the study. We excluded patients with metabolic, endocrine, hepatic or renal diseases. Furthermore, no patient assumed steroid or non-steroidal anti-inflammatory drugs, including statins and beta-blockers or received any transfusion. Patients were divided in two groups each of 12 subjects according to their age. In the first group were allocated patients aged < 61 years (younger group), in the other group were included patients aged > 76 years (older group). Pre-operatively, all patients received antibiotic prophylaxis (1 g of intramuscular cefuroxime). The hernias were classified in type II and III A according to Nyhus classification [11]. Age, gender, anaesthesiologic grading, duration of operation and body weight index are given in Table 1. The only significant difference between the two groups was observed for age, while type and location of hernia, body mass index, anaesthesiologic grading and duration of operation were not significantly different. All patients showed an uncomplicated intra and post-operative course. Both study groups received the standard anaesthetic procedures using a mixture of 40 ml 1% mepivacaine and 20 ml 0.5% bupivacaine plus 2 ml of sodium bicarbonate. All operations were performed through an oblique inguinal incision. The hernia sac, isolated from related structures, was never resected. All patients underwent to tension-free hernioplasty by LH procedure using a polypropylene mesh (Prolene mesh, Ethicon, Somerville, NJ, USA) secured in position by polypropylene staples [9]. The oblique muscle fascia was sutured using Dexon (Davis-Geck, Wayne, NJ, USA). Patients left the hospital 8–24 hours after the operation. Peripheral venous blood samples were collected 24 hours prior to surgery and then 6, 24, and 48 hours post-operatively. The study was approved by the University Hospital Ethics Committee and informed consent was obtained from all subjects.

**Table 1:** Patients characteristics

	**Young**	**Older**
**Age **(years)	51.8 ± 8.9°	82.4 ± 4.7
**Right/Left**	7/5	6/6
**Hernia (Type Nyhus) **II	6	7
III A	6	5
**Body Mass Index **(Kg/m^2^)	26 ± 8	31 ± 11
**Anesthesiologic grading **(ASA) I	8	6
II	4	6
**Duration of the operation **(min)	29 ± 9	31 ± 15
**Hospital discharge **(hours)	16 ± 5	17 ± 4.5
**Complications**	0	0

° Significant differences Young vs. Older p = .01 (t Student 's test).

### Assays

Absolute numbers and percentages of leukocyte blood counts were performed by using ADVIA 120 (Bayer Diagnostics, Munich, Germany). Sera were obtained from blood within 30 min of venipuncture by clotting and centrifugation at 400 g for 10 min. Samples were aliquoted and frozen at-70°C until assay. For the quantitative determination of TNF-α and IL-1β, assays were performed with commercially available kits according to the manufacturer's instructions (R&D Systems Inc., Minneapolis, MN, USA). To standardise our results, in all assays cytokine reference preparations (recombinant human cytokines) were tested. All standards and samples were tested in duplicate. By using these kits, the detection limits were 1.6 pg/ml for TNF-α and 1 pg/ml for IL-1β. High-sensitivity CRP and AAT serum levels were determined with commercial kits by nephelometric analysis (Behring, Vienna, Austria).

### Statistical analysis

Values were given as mean ± SD. T student's test was used to compare the values of young and old subjects. In both groups of patients postoperative values were compared to baseline values by one -way analysis of variance (ANOVA). Differences were considered significant when attained a p value = 0.05.

## Results

**Figure 1:**
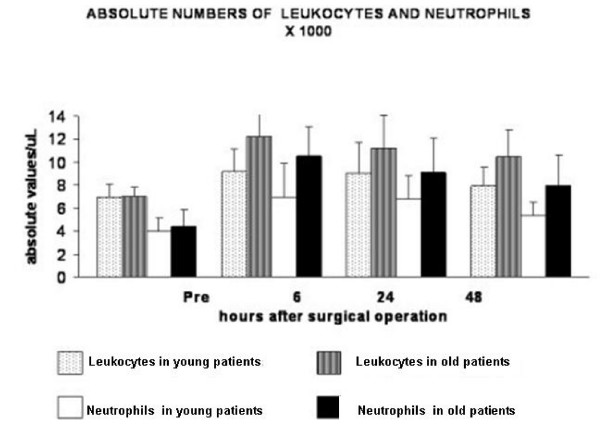
Hernioplasty-caused neutrophilia. Concerning significance between baseline values and post-operative values, in young patients the differences were not significant (ANOVA), whereas in old subjects were significant at all the times (p < 0.001, p < 0.001 and p < 0.01 respectively) (ANOVA). The neutrophilia was significantly higher in old patients, when compared to the young ones, at 6 h and 24 h after the operation (p = 0.02 and p = 0.003 respectively) (t Student's test).

**Figure 2:**
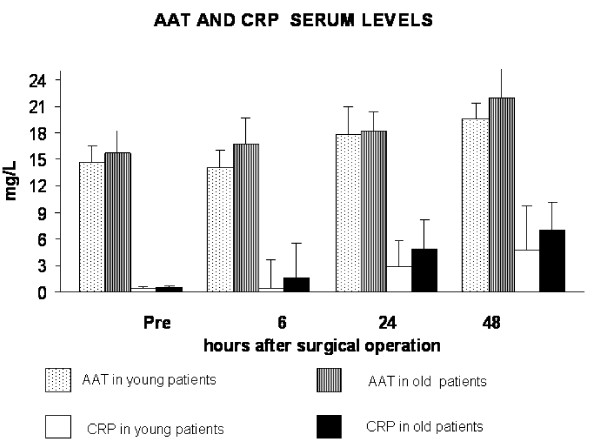
Hernioplasty-caused increase of CRP and AAT values. Concerning AAT, a significant increase of post-operative values respect to baseline ones was observed at 24 h (p < 0.05) and 48 h (p = 0.01 by ANOVA) in young patients, whereas in old subjects they were significant only at 48 h (p < 0.01 by ANOVA). Regarding CRP, in young patients the differences between baseline values and post-operative values were significant only at 24 h (p < 0.05 by ANOVA), whereas in old subjects were significant at 24 (p < 0.05) and 48 hours (p < 0.001 by ANOVA). Besides, the significance was obtained by comparing values of young and old patients at 6 h, 24 h and at 48 h after the operation (p = 0.028, p = 0.05 and p = 0.098 respectively for both by t Student 's test).

**Figure 3:**
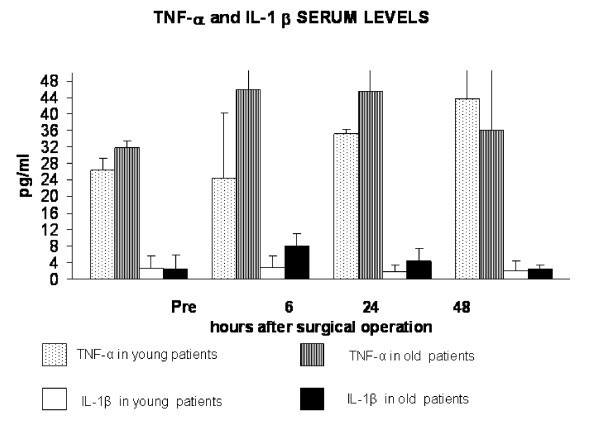
Hernioplasty-caused increase of TNF-α and IL-1β values. A significant increase in TNF-α post-operative values in comparison with the baseline ones, was observed only in young subjects at 48 h after the operation (p < 0.05 by ANOVA). In elderly TNF-α values were not significantly higher when compared with baseline values. They were significantly higher in older patients than in younger at 6 h and 24 h after the operation (p = 0.003 and p= 0.047, respectively by t Student's test). A significant increase of IL-1β post-operative values respect to baseline values was observed only in old patients at 6 h (p < 0.01 by ANOVA) and IL-1β values were significantly higher in older patients than in younger at 6 h after the operation (p = 0.002 by t Student's test).

The analysis of leukocytes counts and of serum values of acute phase proteins and pro-inflammatory cytokines in both groups of young and old patients are shown in Figures 1, 2, 3. As it can be observed, the postoperative values are, on the whole, higher in both groups of subjects when compared to baseline values and they are higher in elderly patients than in the young ones. However, likely due to the relatively small number of patients some discordant results were obtained and significance was not always attained. Regarding leukocytes, the only significant differences between the two groups were observed for neutrophils. Indeed, hernioplasty determined neutrophilia both in young and old patients (Figure 1). However, in young patients, the differences between baseline and postoperative values were not significant by ANOVA, whereas in old subjects were significant at all the times (p < 0.001, p < 0.001 and p < 0.01 respectively by ANOVA) (Figure 1). The neutrophilia was significantly higher in old patients, when compared to the young ones, at 6 h and 24 h after the operation (p = 0.02, p = 0.003 respectively by t Student's test) (Figure 1).

Data on AAT and CRP are presented in Figure 2. Hernioplasty determined an increase of both reactants values in young and old patients. Concerning AAT, a significant increase of post-operative values respect to baseline ones was observed at 24 h (p < 0.05) and 48 h (p < 0.01 by ANOVA) in young subjects, whereas in old subjects they were significant only at 48 h (p < 0.01 by ANOVA) (Figure 2). No significance was obtained by comparing values of young and old patients at all the times (by t Student's test) (Figure 2). Regarding CRP, in young patients the differences between baseline values and post-operative values were significant only at 24 h (p < 0.05 by ANOVA), whereas in old subjects were significant at 24 (p < 0.05) and 48 hours (p < 0.001 by ANOVA) (Figure 2). Besides, significance was obtained by comparing values of young and old patients at 6 h, 24 h and at 48 h after the operation (p = 0.028, p = 0.05 and p = 0.098 respectively by t Student 's test) (Figure 2).

Data on pro-inflammatory cytokines are presented in Figure 3. Hernioplasty determined an increase of TNF-α values both in young and old patients. A significant increase in TNF-α post-operative values in comparison with the baseline ones, was observed only in young subjects at 48 h after the operation (p < 0.05 by ANOVA) (Figure 3). In elderly, TNF-α values were not significantly higher when compared with baseline values (Figure 3), likely because ageing is accompanied by an increase of TNF-α values [4,8]. Accordingly, they were significantly higher in older patients than in younger at 6 h and 24 h after the operation (p = 0.003 and p= 0.047, respectively by t Student's test). A significant increase of IL-1β post-operative values respect to baseline values was observed only in old patients at 6 h (p < 0.01 by ANOVA) and IL-1β values were significantly higher in older patients than in younger at 6 h after the operation (p = 0.002 by t Student's test) (Figure 3).

## Discussion

In this report, as a model of surgical stress, we studied the response to hernioplasty using LH procedure in healthy young and old patients. The model has two major advantages: the possibility of exposing subjects for exactly the same immunological challenge, i.e. the hernioplasty using the same procedure; besides, the uncomplicated disease does not affect pro/anti-inflammatory status of the subject, resulting in a clean in vivo model. Furthermore, to avoid confounding effects linked to post- and pre-menopausal, we studied only male patients.

Several studies have demonstrated that a increased inflammatory activity accompanies ageing. In fact, old and oldest old individuals show by 2–4 fold increased plasma and serum levels of inflammatory mediators such as cytokines and acute phase proteins [4,8]. Accordingly, we performed this study to examine if the APR elicited by surgical intervention differed between old and young individuals. So, we measured the changes in concentrations of leukocytes, of proinflammatory cytokines, including TNF-α and IL-1β, of acute phase proteins such as CRP and AAT in sera from 12 old patients and 12 young patients at preoperative time and at 6, 24, 48 hours postoperatively.

We have selected TNF-α and IL-1β as pro-inflammatory cytokines because they are the first cytokines produced at the site of the surgical trauma by macrophages and monocytes [12,13]. Within the cytokine networking during the surgical trauma-induced APR, IL-6 production is stimulated by the other two cytokines [13]. Besides, in elderly IL-6 plasma levels act as a marker of subclinical cardiovascular diseases [14], depending also on visceral fat amount (Franceschi, personal communication) and on strong genetic control [15]. As regards anti-inflammatory cytokine IL-10, the study of Krabbe et al. [16] on response to endotexemia demonstrates the same change patterns of IL-10 serum levels in elderly and young controls. Furthermore, we have previously demonstrated that IL-10 polymorphisms linked to high cytokine production are significantly increased in healthy oldest old [17]. Taking into account all these things, in the present study, we have focused only on pro-inflammatory cytokines IL-1β and TNF-α. Elderly humans showed prolonged and strong inflammatory activity compared to younger subjects in response to surgical stress, indicating that the APR to surgical stress of elderly humans varies from that of the young, showing initial hyper-reactivity and a delayed termination of the response. The more extensive inflammation in elderly patients was not due to longer and more complicated surgery, increased fat mass or increased morbidity, since no differences were observed between the two groups (Table 1). So, it was due to ageing per se. However, as stated in the results, some discordant findings have been obtained. That might due to the relatively small sample. On the other hand, it may be argued that a Bonferroni-type adjustment should be performed to correct for the testing of the multiple variables under study. However, this correction is too stringent and has the potential to ignore important observations [18].

Pneumococcal infections also determined higher levels of TNF-α in blood of old patients when compared to those of young patients. Furthermore, old subjects show a more rapid increase in CRP levels than that detected in the young group [16]. Other studies have reported increased circulating levels of TNF-α and acute-phase proteins, following an intravenous bolus of endotoxin (2 ng/kg) in elderly persons as well as increased unstimulated production of IL-1β, IL-6 and IL-1receptor antagonist in vitro [4,8].

In conclusion, our results demonstrate that the APR to surgical stress of elderly humans varies from that of the young, showing initial hyper-reactivity and a delayed termination of the response. That might be related to the well-documented basal low-grade pro-inflammatory activity in the elderly respect to young people. It is possible that a wide range of factors contributes to this basal low-grade inflammation, including an increased amount of fat tissue, smoking, sub-clinical infections and chronic disorders such as cardiovascular diseases and Alzheimer's disease [19]. Alternatively, the pro-inflammatory status observed in older persons might depend on the chronic antigenic stress which bombards the innate immune system thorough out life since old people have to cope with a lifelong antigenic burden encompassing several decades of evolutionary unpredictable antigenic exposure [17]. In fact, a major consequence of chronic exposure to antigens is the progressive activation of macrophages and related cells in most organs and tissues of the body [17,20].

However, the clinical significance of our data is not clear. It is not known whether a causal relationship exists between the cytokine and acute phase response and the increases in susceptibility to post-operative complications observed in aged patients. On the other hand, pro-inflammatory cytokines have been claimed to play an important role in the pathogenesis of postoperative complications [5,21]. Accordingly, our model of surgical stress can suggest that oldest old people is more prone to postoperative complications because is able to produce higher amount of pro-inflammatory cytokines.
